# How policy implementation shapes the impact of U.S. food assistance policies: the case study of the Child and Adult Care Food Program

**DOI:** 10.3389/frhs.2023.1286050

**Published:** 2023-11-02

**Authors:** Erica L. Kenney, Mary Kathryn Poole, Natasha Frost, Kelsey Kinderknecht, Rebecca S. Mozaffarian, Tatiana Andreyeva

**Affiliations:** ^1^ Department of Nutrition, Harvard T.H. Chan School of Public Health, Boston, MA, United States; ^2^ Seed 2 Roots LLC, Mankato, MN, United States; ^3^ Department of Agricultural and Resource Economics, Rudd Center for Food Policy and Health, University of Connecticut, Storrs, CT, United States

**Keywords:** policy, implementation science, nutrition assistance, childcare, public health

## Abstract

Much of the chronic disease burden in the U.S. population can be traced to poor diet. There has been a sustained focus on influencing children's diets and encouraging healthier eating habits by changing policies for what foods and beverages can be served to children through large federally-funded nutrition assistance programs. Yet without attention to how nutrition policies are implemented, and the surrounding context for these policies, these policy changes may not have the intended results. In this perspective, we used Bullock et al.'s (2021) Process Model of Implementation from a Policy Perspective to analyze how the complexities of the implementation process of large-scale nutrition policies can dilute potential health outcomes. We examine the Child and Adult Care Food Program (CACFP), a federal program focused on supporting the provision of nutritious meals to over 4 million children attending childcare, as a case study. We examine how the larger societal contexts of food insecurity, attitudes towards the social safety net, and a fragmented childcare system interact with CACFP. We review the “policy package” of CACFP itself, in terms of its regulatory requirements, and the various federal, state, and local implementation agencies that shape CACFP's on-the-ground implementation. We then review the evidence for how each component of the CACFP policy implementation process impacts uptake, costs, feasibility, equity, and effectiveness at improving children's nutrition. Our case study demonstrates how public health researchers and practitioners must consider the complexities of policy implementation processes to ensure effective implementation of nutrition policies intended to improve population health.

## 1. Introduction

The United States (U.S.) faces substantial public health challenges related to poor nutrition. Diet-related chronic diseases (1)—including heart disease, stroke, type II diabetes mellitus, and certain types of cancer (2, 3)– are experienced by most U.S. adults, contributing to poor health and early mortality (4, 5). Moreover, inequities in access to affordable, nutritious foods have resulted in socioeconomic and racial/ethnic disparities in diet quality (6–8). Income and race are also closely linked with a higher risk of food insecurity (9), which further increases the risk of both poor diet and cardiometabolic diseases (10, 11). These problems start in childhood (2, 12, 13).

To address these population-wide challenges, policymakers have leveraged federal child nutrition assistance programs, such as the Special Supplemental Nutrition Program for Women, Infants, and Children (WIC), the National School Lunch and Breakfast Programs (NSLP/SBP), and the Child and Adult Care Food Program (CACFP), as policy levers for achieving public health nutrition goals (14–17). These programs, which provide financial support to improve food security and access for Americans, especially those with lower incomes, show promise for improving diet quality and reducing health inequities. In recent years, efforts to bring minimum nutrition standards for WIC and NSLP/SBP in line with current dietary science have resulted in substantial improvements in the diet quality and chronic disease risk of program participants (18–24), suggesting that policy changes to these programs can be a promising approach to population health. However, similar updates to CACFP appear to have had less strong effects (25).

In this perspective, we use a conceptual framework of policy implementation, developed by Bullock et al. (26), to outline the challenges in leveraging federal nutrition policies as public health interventions. We specifically examine CACFP, which provides reimbursements to child and adult daycare providers to support serving meals and snacks meeting basic nutritional standards (27), as a case study (we focus here solely on childcare providers and child-level outcomes, given that these are the majority beneficiaries of CACFP). Given that CACFP appears to have less consistently strong impacts on child nutrition compared to other federal nutrition programs, we seek to understand how its policy implementation process may explain why.

## 2. Conceptual framework for the analysis

Bullock et al.'s Process Model of Policy Implementation (2021) (26) posits that policies are first borne out of a larger context of existing ideas, interests, and other external factors that determine how a problem is defined and whether it is addressed by policy in the first place. This brings about the development of a policy package, a collection of strategies like regulations or statutes, economic incentives, voluntary guidelines, or information campaigns. The implementation process of the policy package then flows through implementing organizations to street-level bureaucrats to recipients. To evaluate policy implementation, outcomes at several levels can be considered, including implementation outcomes (28) (e.g., fidelity, uptake, acceptability, costs, feasibility, sustainability), service outcomes (e.g., effectiveness, equity, efficiency), recipient outcomes (e.g., changes in actual recipient behavior, satisfaction), and policy/system level outcomes (e.g., reductions in food insecurity at a population level).

**Figure 1:**
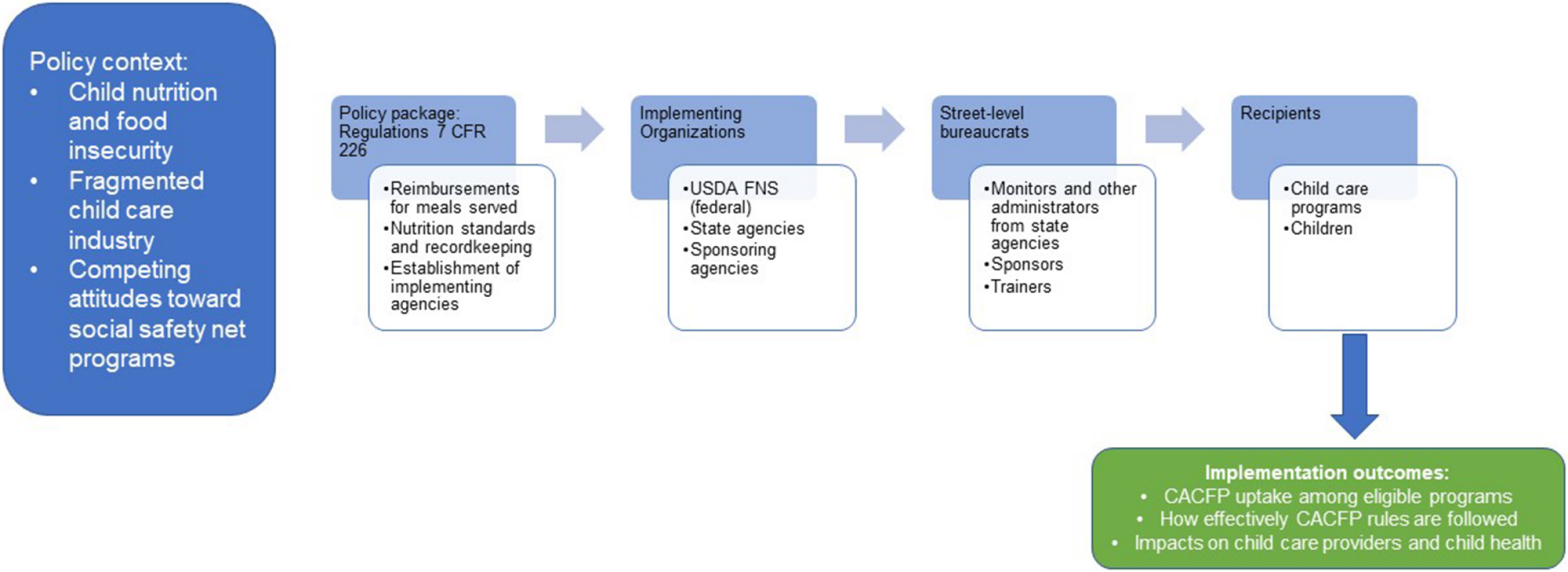
Process model of policy implementation applied to the Child and Adult Care Food Program.

Figure 1 presents an adaptation of Bullock et al.'s model for this paper's analysis of CACFP. In the following sections, we explore each of the key phases of the implementation process described in the Process Model—context, developing the policy package, processing through implementing organizations, street-level bureaucrats, and recipients, and finally outcomes—for CACFP.

## 3. CACFP's implementation process and how its attributes determine implementation outcomes

### 3.1. Outer context: child food insecurity, child development, the childcare industry, and attitudes towards social safety net programs

CACFP exists in a larger context related to child health, and specifically children's nutrition, in the U.S. Food insecurity currently affects 12.5% of households with children in the U.S. (9). Additionally, even for children not experiencing food insecurity, the nutritional quality of foods available to and consumed by children is often poor, with high amounts of inexpensive, highly palatable ultraprocessed foods (29) and inadequate consumption of vegetables, whole grains, and lean protein sources (12, 30). Several decades ago, a key dietary concern was inadequate intake of essential micronutrients; more recently, overconsumption of foods and beverages that can lead to excess weight gain for healthy growth has become a concern for children (31–36).

While social safety net programs have been designed to mitigate these public health nutrition challenges for households with low incomes, there are disagreements on how comprehensive the programs should be (37).CACFP falls within this challenging context.

This struggle can be seen in CACFP's history (38). CACFP's roots lie in a federal pilot program called the Special Food Service Program for Children, started in 1968, at a time in U.S. history when the social safety net was being radically expanded through President Lyndon B. Johnson's “War on Poverty”. This program was expanded and formalized into CACFP across the 1970s and 1980s providing childcare providers with resources to serve free rather than including meal costs in tuition or not serving meals at all (38). However, in the Personal Responsibility and Work Opportunity Reconciliation Act of 1996—a law which made several safety net programs more difficult to access—CACFP was modified to cut costs. This law reduced the number of meals for which providers could receive reimbursements and introduced an income-based tiering system for reimbursements that reduced the overall financial support most providers could receive and also introduced additional administrative burden (39) to the program, as it necessitated providers' collection of income information for the families they served (38). Although future legislative actions allowed for relatively small expansions of the program after this, little changed about CACFP until 2017, when the nutrition standards for CACFP meals were updated as part of the Healthy, Hunger Free Kids Act of 2010 (40, 41). A report by the National Academies of Medicine in 2011 suggested sweeping changes were needed to bring CACFP meal pattern standards in line with dietary science regarding child health and development (38), yet no additional federal funding was appropriated to support these changes (41).

An additional complexity is how fractured and underfunded the childcare system is in the U.S. Unlike most other economically developed countries; the U.S. has no universal public system of childcare (42). The childcare industry mostly relies on tuition payments from families and depressed wages for childcare workers in order to function (43). The industry has been referred to as a “textbook example” of a broken market (44): parents have to pay so much in tuition that it prohibits many from participating in the workforce at all, educators and other staff are underpaid, and owners are often barely able to keep the programs breaking even. Childcare providers and educators, who face substantial physical and mental health challenges personally (45), thus often face multiple intense challenges related to simply maintaining operations and adhering to their state's existing regulatory requirements. In this organizational context, participating in CACFP, or even serving meals in the first place, can add an additional layer of complexity to an already challenging situation.

### 3.2. Policy package: what are the regulations and unofficial rules that make up CACFP?

The regulations for CACFP (7 CFR Part 226) (46) outline minimum requirements for foods and beverages served for various age groups (see Table 1). Participating providers can receive reimbursements for up to three meals and/or snacks. The reimbursement amount for centers varies according to the household income status of the recipient child; reimbursements for family childcare providers (who provide care in their home to a smaller group of children compared to childcare centers) vary based on neighborhood-level income metrics (using either Census or local school meals data) (46). To participate in CACFP, providers must prove that they either have a nonprofit status or that they serve at least 25% of children from low income households (47) and must prove financial viability (46). They must submit paperwork on the foods and beverages served for each meal, the amounts served, and child attendance as well as documentation of receipts and compliance with civil rights law. Providers and key staff participate in annual trainings and periodic monitoring visits from state auditors to assess compliance (46).

**Table 1:** CACFP meal pattern requirements and reimbursements per meal, 2023-2024.

	Required components	Reimbursement for centers	Reimbursement for daycare homes^a^
Breakfast	Fluid milk (4 oz for ages 1–2, 6 oz for ages 3–5, 8 oz for ages 6 and up) Vegetables, fruits, or both (1/4 cup for ages 1–2, ½ cup for ages 3 and up) Grains (1/2 oz for ages 1–5, 1 ounce for ages 6 and up)	Free: $2.28 Reduced price: $1.98 Paid: $0.38	Tier I: $1.65 Tier II: $0.59
Lunch and supper	Fluid milk (4 oz for ages 1–2, 6 oz for ages 3–5, 8 oz for ages 6 and up) Meat/meat alternates (serving sizes vary by type, generally between 1 and 2 oz) Vegetables (1/8 cup for ages 1–2, ¼ cup for ages 3–5, ½ cup for ages 6 and up) Fruits (1/8 cup for ages 1–2, ¼ cup for ages 3 and up) Grains (1/2 oz for ages 1–5, 1 ounce for ages 6 and up)	Free: $4.25 Reduced price: $3.85 Paid: $0.40	Tier I: $3.12 Tier II: $1.88
Snack	Select two of the components listed above for lunch, with larger serving sizes for fruits/vegetables and smaller serving sizes for meat/meat alternates	Free: $1.17 Reduced price: $0.58 Paid: $0.10	Tier I: $0.93 Tier II: $0.25
Other nutrition requirements:	Milk must be unflavored, whole milk for children aged one, unflavored low fat or skim milk for children ages 2–5, and unflavored or flavored low fat milk for ages 6 and up. Yogurt must contain no more than 23 g total sugars per 6 ounces. 100% juice may be used to meet a vegetable or fruit requirement at only one meal each day Vegetables may be used to meet entire fruit + vegetable requirement at lunch At least one serving of whole grains is required each day Breakfast cereals cannot contain >6 g sugar per dry ounce		

Sources: Available at: https://www.fns.usda.gov/cacfp/meals-and-snacks; Available at: https://www.fns.usda.gov/cacfp/reimbursement-rates.

^a^ Amounts presented are for contiguous U.S. states; amounts are higher for Alaska, Hawaii, and U.S. territories.

### 3.3. Implementing organizations and street-level bureaucrats: who implements CACFP?

CACFP is a federal program that is administered by state agencies. At the federal level, the U.S. Department of Agriculture Food and Nutrition Service (USDA FNS) tracks participation, issues guidance to state agencies on how to comply with regulations, releases technical assistance tools (like recipes and worksheets), and commissions program evaluations. State agencies, however—typically education or public health agencies—are the organizations that are responsible for most administrative activities, including approving and onboarding new participating providers, disbursing reimbursements, monitoring compliance, providing technical assistance, and maintaining participation records (48).

The state agency is also responsible for registering and working with sponsoring agencies or sponsors, which also support implementation. Family childcare providers are required to work with sponsors so that these agencies can complete some of their administrative paperwork and provide technical assistance; depending on the state, some centers can also work with sponsors, or operate independently (49).

The “street-level bureaucrats” involved in implementing CACFP on the ground are state agency staff responsible for auditing participating programs and sponsors, as well as sponsors themselves, who help participating providers comply with program rules.

### 3.4. Recipients: childcare programs and children

One unusual aspect of CACFP is that childcare providers can be thought of both as the recipients of the program—they receive the reimbursements for the meals they serve—and also a type of “street-level bureaucrat” as they are implementing the program day-to-day: planning menus, obtaining or preparing food, gathering families' income-eligibility information, participating in training, maintaining paperwork, and submitting to monitoring visits. The other recipients of the program are the children receiving the meals and snacks.

### 3.5. Outcomes: what do we know about CACFP's impact?

#### 3.5.1. Implementation outcomes

These include acceptability, adoption, appropriateness, costs, feasibility, fidelity, uptake (penetration), and sustainability (26, 28). We present evidence for four of these constructs with existing evidence below.

##### 3.5.1.1. Penetration

While the USDA estimates that CACFP served up to 4.6 million children in 2021, it does not track the percentage of eligible programs that participate. A recent analysis of state administrative records, however, estimated that only about a third of licensed childcare centers participate in CACFP nation-wide, with large variability across states (16%–86%) (50).

##### 3.5.1.2. Fidelity

Studies of the degree to which programs adhere to CACFP's regulatory standards generally suggest that programs meet the standards most of the time, but not perfectly (25, 51, 52).

##### 3.5.1.3. Feasibility

Providers have consistently reported that CACFP is difficult to use, citing the burden of paperwork, inadequate staff, insufficient reimbursements, mismatch of the meal pattern standards with child preferences, and inflexibility of the standards for cultural foods as being key barriers to feasibility (53–56).

##### 3.5.1.4. Cost

Although CACFP reimburses providers for each qualifying meal and snack served (as described above), many studies have found that the reimbursement is not adequate. While some studies have found that the reimbursement covers the costs of food (57–59), studies have also found that the reimbursement is not adequate for supporting foods with more variety that can improve diet quality and support children's preferences (60, 61), and that the reimbursement is not adequate to cover labor costs (59).

#### 3.5.2. Service outcomes

Include efficiency, safety, effectiveness, equity, client-centeredness, and timeliness (26, 28). We present analysis for four of these constructs with existing evidence below.

##### 3.5.2.1. Efficiency

As described above, there are substantial monitoring activities involved that make CACFP's efficiency questionable. Daily meal and attendance counts, menu planning, managing food receipts to demonstrate compliance, reviewing food labels to assess whether foods are creditable, and also the work involved in soliciting and organizing income-eligibility paperwork from parents all contribute to substantial administrative burden (39).

##### 3.5.2.2. Effectiveness

A recent systematic review of studies of the impact of CACFP on the nutritional quality of meals served in childcare programs found mixed evidence overall for a beneficial impact of CACFP, partly due to a lack of rigorous, large-scale studies. Existing studies either find null associations between CACFP and nutritional quality or typically very small positive associations (62).

##### 3.5.2.3. Equity

It is unknown whether CACFP is accessed inequitably. There are concerns, however, in how programs located in food deserts—which often track with both rural locale and with areas subjected to racialized segregation (63, 64)—may have difficulty accessing foods compliant with CACFP meal pattern standards. Additionally, the administrative burden of this program itself may produce inequities. Childcare providers serving higher income families can opt out of CACFP. Such programs can either have parents provide meals themselves, or pay extra in tuition to cover meal service costs. Therefore, the administrative burden is borne by providers serving children from households with lower income.

#### 3.5.3. Recipient outcomes

##### 3.5.3.1. Providers

It is unclear the extent to which CACFP benefits providers themselves; most studies evaluate the impacts of CACFP on childcare program practices and policies. For example, it is unclear whether CACFP actually helps providers financially so that they have less business challenges or are able to keep program tuition lower. It is also unclear whether CACFP helps with providers' own health and wellness. Notably, despite the fact that childcare teachers are strongly encouraged to sit and eat with children during mealtimes, meals for teachers are not reimbursable through CACFP currently.

##### 3.5.3.2. Children

Similar to what has been found in evaluations of CACFP's impact on childcare program-level food practices and policies, evidence for a beneficial impact of CACFP on child-level outcomes, including diet quality, food security, and healthy weight, are mixed, with studies either finding null or very slightly positive associations (62).

#### 3.5.4. Policy outcomes

Overall, it is unclear whether CACFP has population-level impacts on childcare meal quality or child health.

## 4. Discussion

Nutrition policies, especially federal nutrition assistance programs, show enormous potential for supporting children's nutrition on a population level. CACFP could be particularly promising given that it focuses on supporting healthy meals for young children, who are at a crucial stage of development. Yet despite its promise, it has not been shown to have strong impacts on child food insecurity, growth, or diet quality (62) While it is often suggested that CACFP participants need more training or technical assistance to support better adoption of CACFP, the analysis above suggests that simply providing training or technical assistance is not enough; rather, we argue that several key misalignments between CACFP's policy implementation process and the current structure of the childcare industry have contributed to weaker impacts. These include:

### 4.1. Fractured childcare industry and conflict over resources for safety net programs

The daily challenges that childcare providers face in maintaining operations—low wages, high staff turnover, high operating costs, and the need to comply with multiple regulations outside of food-related rules—may make participation in CACFP infeasible for many programs; they just may not have the bandwidth given the current structure of childcare. While increasing operational and financial support given through CACFP could increase its feasibility for programs, as well as providing more support and structure to the childcare industry in general, this would require expansions of the existing social safety net that are controversial in the current political climate.

### 4.2. Insufficient financial support for providers to effectively implement the program

While existing reimbursements may cover food costs on average, they do not appear to be adequate for covering the cost of the labor needed to complete CACFP's administrative requirements or to plan and prepare meals. They also may not be adequate for supporting a variety of foods that can fit children's preferences or help towards introducing children to new foods, leaving providers with a situation where they are repeating the same few meals and reducing satisfaction with the program. Reimbursements that fairly cover the costs of labor and can support a truly healthful food service—with the provision of a variety of foods that meet CACFP's nutritional standards and children's preferences—are needed.

### 4.3. Inadequate implementation structure for some programs

An implementation structure more similar to that of the NSLP/SBP—where there are agencies with dedicated staff for overseeing compliance paperwork, planning meals, and preparing and serving meals—could be helpful. For many childcare providers, especially those without a sponsor, participating in CACFP would be akin to asking school principals and teachers to add school meal compliance paperwork and food service to their workloads. Sponsoring agencies help support family childcare providers and some centers in overseeing administrative duties; perhaps a more robust role for these agencies, with support available for more center-based programs and more help with the meal planning and food preparation tasks necessary for participation, could be a solution.

Additionally, increasing communication across levels of implementation (federal, state, sponsor, provider) is needed. The agency involved in setting policy—USDA—is far removed from the day-to-day activities involved in implementation. One implication of this is that some of the policy memorandums that USDA provides to try to support implementation, as well as informational resources designed to help providers comply, may be out of sync with what providers need. For example, one co-author, who is involved in providing food service for CACFP-participating programs, has found that USDA's example recipes often include foods that are too expensive (like nuts or dried fruits) and/or foods that are not creditable for that dish. Communication between state agencies and food vendors could be further developed, rather than relying on childcare centers to navigate those communications. Finally, supporting newly-formed childcare providers in the transition of opening could be a useful investment to ensure the food programs are a support rather than a burden to newly-formed business enterprises.

## 5. Conclusions

Policymakers and others involved in policy formulation and implementation processes should consider strategies to reshape CACFP's implementation to better fit the existing context of childcare in the U.S.—not only through more robust financial support, but also through perhaps a reconsideration of what administrative paperwork is truly necessary for program participation and a retooling of existing implementation supports, like training, technical assistance, and meal planning, that are available to childcare programs. Meanwhile, as this analysis demonstrates, we suggest that researchers, policymakers, and public health practitioners who want to leverage food policies to promote public health nutrition must go beyond focusing only on requiring the provision of foods and beverages in line with dietary science—we must also carefully consider the context in which these policies operate, and the implementation process that can determine their success.

## Data Availability

The original contributions presented in the study are included in the article/Supplementary Material, further inquiries can be directed to the corresponding author.

## References

[1] GBD 2017 Diet Collaborators. Health effects of dietary risks in 195 countries, 1990-2017: a systematic analysis for the global burden of disease study 2017. Lancet. (2019) 393(10184):1958–72. 10.1016/S0140-6736(19)30041-8 30954305 PMC6899507

[2] Liu J Rehm CD Onopa J Mozaffarian D. Trends in diet quality among youth in the United States, 1999-2016. JAMA. (2020) 323(12):1161–74. 10.1001/jama.2020.0878 32207798 PMC7093765

[3] Rehm CD Peñalvo JL Afshin A Mozaffarian D. Dietary intake among US adults, 1999–2012. JAMA. (2016) 315(23):2542–53. 10.1001/jama.2016.7491 27327801 PMC6287627

[4] Centers for Disease Control and Prevention. About chronic diseases (2023). Available at: https://www.cdc.gov/chronicdisease/about/index.htm (Accessed July 31, 2023).

[5] Centers for Disease Control and Prevention. Leading causes of death (2023). Available at: https://www.cdc.gov/nchs/fastats/leading-causes-of-death.htm (Accessed July 31, 2023).

[6] Odoms-Young AM. Examining the impact of structural racism on food insecurity: implications for addressing racial/ethnic disparities. Fam Commun Health. (2018) 41:S3–6. 10.1097/FCH.0000000000000183 PMC5823283 29461310

[7] Cooksey Stowers K Jiang Q Atoloye AT Lucan S Gans K. Racial differences in perceived food swamp and food desert exposure and disparities in self-reported dietary habits. Int J Environ Res Public Health. (2020) 17(19):7143. 10.3390/ijerph17197143 33003573 PMC7579470

[8] Carroll-Scott A Gilstad-Hayden K Rosenthal L Peters SM McCaslin C Joyce R Disentangling neighborhood contextual associations with child body mass index, diet, and physical activity: the role of built, socioeconomic, and social environments. Soc Sci Med. (2013) 95:106–14. 10.1016/j.socscimed.2013.04.003 23642646 PMC4058500

[9] Coleman-Jensen A Rabbitt MP Gregory CA Singh A. Household food security in the United States. Econ Res Rep. (2022) 155.

[10] Seligman HK Berkowitz SA. Aligning programs and policies to support food security and public health goals in the United States. Annu Rev Public Health. (2019) 40:319–37. 10.1146/annurev-publhealth-040218-044132 30444684 PMC6784838

[11] Te Vazquez J Feng SN Orr CJ Berkowitz SA. Food insecurity and cardiometabolic conditions: a review of recent research. Curr Nutr Rep. (2021) 10(4):243–54. 10.1007/s13668-021-00364-2 34152581 PMC8216092

[12] Welker EB Jacquier EF Catellier DJ Anater AS Story MT. Room for improvement remains in food consumption patterns of young children aged 2–4 years. J Nutr. (2018) 148(9S):1536S–46S. 10.1093/jn/nxx053 29878237 PMC6126636

[13] Larson N Story M. Barriers to equity in nutritional health for US children and adolescents: a review of the literature. Curr Nutr Rep. (2015) 4(1):102–10. 10.1007/s13668-014-0116-0

[14] Mozaffarian D Angell SY Lang T Rivera JA. Role of government policy in nutrition—barriers to and opportunities for healthier eating. Br Med J. (2018) 361:k2426. 10.1136/bmj.k2426 29898890 PMC5997034

[15] Bleich SN Moran AJ Vercammen KA Frelier JM Dunn CG Zhong A Strengthening the public health impacts of the supplemental nutrition assistance program through policy. Annu Rev Public Health. (2020) 41:453–80. 10.1146/annurev-publhealth-040119-094143 32237988

[16] Koleilat M Whaley SE Esguerra KB Sekhobo JP. The role of WIC in obesity prevention. Curr Pediatr Rep. (2017) 5:132–41. 10.1007/s40124-017-0135-6

[17] World Health Organization. Assessing the existing evidence base on school food and nutrition policies: a scoping review. (2021).

[18] Daepp MIG Gortmaker SL Wang YC Long MW Kenney EL. WIC Food package changes: trends in childhood obesity prevalence. Pediatrics. (2019) 143(5):e20182841. 10.1542/peds.2018-2841 30936251 PMC6565338

[19] Tester JM Leung CW Crawford PB. Revised WIC food package and childrens diet quality. Pediatrics. (2016) 137(5):e20153557. 10.1542/peds.2015-3557 27244804 PMC4845874

[20] Schultz DJ Byker Shanks C Houghtaling B. The impact of the 2009 special supplemental nutrition program for women, infants, and children food package revisions on participants: a systematic review. J Acad Nutr Diet. (2015) 115(11):1832–46. 10.1016/j.jand.2015.06.381 26276067

[21] Kenney EL Barrett JL Bleich SN Ward ZJ Cradock AL Gortmaker SL. Impact of the healthy, hunger-free kids act on obesity trends. Health Aff (Millwood). (2020) 39(7):1122–9. 10.1377/hlthaff.2020.00133 32634356 PMC7961790

[22] Mozer L Johnson DB Podrabsky M Rocha A. School lunch entrees before and after implementation of the healthy, hunger-free kids act of 2010. J Acad Nutr Diet. (2019) 119(3):490–9. 10.1016/j.jand.2018.09.009 30473488

[23] Kinderknecht K Harris C Jones-Smith J. Association of the healthy, hunger-free kids act with dietary quality among children in the US national school lunch program. JAMA. (2020) 324(4):359–68. 10.1001/jama.2020.9517 32721008 PMC7388023

[24] Johnson DB Podrabsky M Rocha A Otten JJ. Effect of the healthy hunger-free kids act on the nutritional quality of meals selected by students and school lunch participation rates. JAMA Pediatr. (2016) 170(1):e153918. 10.1001/jamapediatrics.2015.3918 26747076

[25] Andreyeva T Mozaffarian RS Kenney EL. Updated meal patterns in the child and adult care food program and changes in quality of food and beverages served: a natural experimental study. Nutrients. (2022) 14(18):3786. 10.3390/nu14183786 36145161 PMC9505753

[26] Bullock HL Lavis JN Wilson MG Mulvale G Miatello A. Understanding the implementation of evidence-informed policies and practices from a policy perspective: a critical interpretive synthesis. Implement Sci. (2021) 16:1–24. 10.1186/s13012-021-01082-7 33588878 PMC7885555

[27] United States Department of Agriculture. Child and adult care food program (CACFP) (2018). Available at: https://www.fns.usda.gov/cacfp/child-and-adult-care-food-program

[28] Proctor EK Landsverk J Aarons G Chambers D Glisson C Mittman B. Implementation research in mental health services: an emerging science with conceptual, methodological, and training challenges. Adm Policy Ment Health. (2009) 36(1):24–34. 10.1007/s10488-008-0197-4 19104929 PMC3808121

[29] Wang L Steele EM Du M Pomeranz JL O’Connor LE Herrick KA Trends in consumption of ultraprocessed foods among US youths aged 2–19 years, 1999–2018. JAMA. (2021) 326(6):519–30. 10.1001/jama.2021.10238 34374722 PMC8356071

[30] Banfield EC Liu Y Davis JS Chang S Frazier-Wood AC. Poor adherence to US dietary guidelines for children and adolescents in the national health and nutrition examination survey population. J Acad Nutr Diet. (2016) 116(1):21–7. 10.1016/j.jand.2015.08.010 26391469 PMC4698034

[31] Centers for Disease Control and Prevention. Childhood overweight and obesity (2022). Available at: https://www.cdc.gov/obesity/childhood/index.html (Accessed August 1, 2023).

[32] World Health Organization. Commission on ending childhood obesity: facts and figures on childhood obesity (2017). Available at: https://www.who.int/end-childhood-obesity/facts/en/ (Accessed August 1, 2023).

[33] Must A Strauss RS. Risks and consequences of childhood and adolescent obesity. Int J Obes Relat Metab Disord. (1999) 23(Suppl 2):S2–11. 10.1038/sj.ijo.0800852 10340798

[34] Ward ZJ Long MW Resch SC Giles CM Cradock AL Gortmaker SL. Simulation of growth trajectories of childhood obesity into adulthood. N Engl J Med. (2017) 377(22):2145–53. 10.1056/NEJMoa1703860 29171811 PMC9036858

[35] May AL Kim SA Sherry B Blanck HM. Childhood obesity task forces established by state legislatures, 2001-2010. Prev Chronic Dis. (2013) 10:E144. 10.5888/pcd10.120153 23987250 PMC3760080

[36] Geserick M Vogel M Gausche R Lipek T Spielau U Keller E Acceleration of BMI in early childhood and risk of sustained obesity. N Engl J Med. (2018) 379(14):1303–12. 10.1056/NEJMoa1803527 30281992

[37] Pew Research Center. Views of the economic system and social safety net (2019). Available at: https://www.pewresearch.org/politics/2019/12/17/views-of-the-economic-system-and-social-safety-net/#long-term-opinion-trends-views-of-the-social-safety-net-and-nations-economic-system (Accessed August 1, 2023).

[38] Murphy SP Yaktine AL West Suitor C Moats S. Child and adult care food program: aligning dietary guidance for all. In: Murphy SP Yaktine AL West Suitor C Moats S, editors. Child and adult care food program: Aligning dietary guidance for all. Washington: National Academies Press (2010). p. xiv+258. doi: 10.17226/12959. 24983044

[39] Herd P Moynihan DP. Administrative burden: policymaking by other means. New York, NY: Russell Sage Foundation (2019).

[40] Healthy, Hunger-Free Kids Act of 2010, Public Law 296, U.S. Statutes at Large 124 (2010). p. 3183–266.

[41] United States Department of Agriculture F and NS. Child and adult care food program: meal pattern revisions related to the healthy, hunger-free kids act of 2010. Washington, DC (2015). Available at: Available from: Available at: https://www.federalregister.gov/articles/2015/01/15/2015-00446/child-and-adult-care-food-program-meal-pattern-revisions-related-to-the-healthy-hunger-free-kids-act 27116762

[42] U.S. Department of the Treasury. The economics of child care supply in the United States. Washington, DC: U.S. Department of Treasury (2021).

[43] Child Care Aware of America. Demanding change: repairing our child care system (2022). Available at: https://info.childcareaware.org/hubfs/2022-03-FallReport-FINAL%20(1).pdf (Accessed August 1, 2023).

[44] Yellen J. Remarks by secretary of the treasury Janet L. Yellen on shortages in the child care system (2021). Available at: https://home.treasury.gov/news/press-releases/jy0355 (Accessed August 1, 2023).

[45] Lessard LM Wilkins K Rose-Malm J Mazzocchi MC. The health status of the early care and education workforce in the USA: a scoping review of the evidence and current practice. Public Health Rev. (2020) 41(1):2. 10.1186/s40985-019-0117-z 31934495 PMC6950818

[46] https://www.ecfr.gov/current/title-7/subtitle-B/chapter-II/subchapter-A/part-226.

[47] United States Department of Agriculture Food and Nutrition Service. Final rule: for-profit center participation in the CACFP (2006). Available at: https://www.fns.usda.gov/cacfp/fr-102306 (Accessed August 1, 2023).

[48] United States Department of Agriculture Food and Nutrition Service. Child and adult care food program: state agency (2021). Available at: https://www.fns.usda.gov/cacfp/state-agency (Accessed August 1, 2023).

[49] United States Department of Agriculture Food and Nutrition Service. Child and adult care food program: program operator (2021). Available at: https://www.fns.usda.gov/cacfp/program-operator (Accessed August 1, 2023).

[50] Andreyeva T Moore TE da Cunha Godoy L Kenney EL. Federal nutrition assistance for young children: under-utilized and unequally accessed. Am J Prev Med. (2023) 13:S0749-3797(23)00354-9. 10.1016/j.amepre.2023.09.008 PMC11000260 37709155

[51] Chriqui JF Leider J Schermbeck RM Sanghera A Pugach O. Changes in child and adult care food program (CACFP) practices at participating childcare and education centers in the United States following updated national standards, 2017–2019. Nutrients. (2020) 12(9):2818. 10.3390/nu12092818 32942598 PMC7551123

[52] Dave JM Cullen KW. Foods served in child care facilities participating in the child and adult care food program: menu match and agreement with the new meal patterns and best practices. J Nutr Educ Behav. (2018) 50(6):582–8. 10.1016/j.jneb.2018.01.010 29475767 PMC5995659

[53] Earnesty D Mphwanthe G Rau K Weatherspoon L. A qualitative study: perceived barriers and facilitators to nutrition standard adherence by in-home childcare providers. J Acad Nutr Diet. (2022) 122(4):786–96.e4. 10.1016/j.jand.2021.08.104 34411786

[54] Jana B Loefstedt K Vu M Ward D Erinosho T. “It has a lot to do with the cumbersome paperwork”: barriers and facilitators of center-based early care and education program participation in the child and adult care food program. J Acad Nutr Diet. (2023) 123(8):1173–1186.e1. 10.1016/j.jand.2023.03.014 36990428

[55] Andreyeva T Sun X Cannon M Kenney EL. The child and adult care food program: barriers to participation and financial implications of underuse. J Nutr Educ Behav. (2022) 54(4):327–34. 10.1016/j.jneb.2021.10.001 34865970

[56] Lee DL Gurzo K Yoshida S Homel Vitale E Hecht K Ritchie LD. Compliance with the new 2017 child and adult care food program standards for infants and children before implementation. Child Obes. (2018) 14(6):393–402. 10.1089/chi.2018.0092 30199288 PMC6150931

[57] Poole MK Cradock AL Kenney EL. Implementing the new child and adult care food program’s nutrition standards in Boston. Prev Chronic Dis. (2020) 17:E44. 10.5888/pcd17.190426 32553072 PMC7316414

[58] Poole MK Cradock AL Kenney EL. Changes in foods served and meal costs in Boston family child care homes after one year of implementing the new child and adult care food program nutrition standards. Nutrients. (2020) 12(9):2817. 10.3390/nu12092817 32942588 PMC7551429

[59] Logan CW Connor P LeClair L Patlan K Glenn M Stidsen C Study of nutrition and activity in childcare settings. Alexandria, VA (2021). Available at: https://fns-prod.azureedge.us/sites/default/files/resource-files/SNACS-AppendixE.pdf (Accessed August 1, 2023).

[60] Monsivais P Johnson DB. Improving nutrition in home child care: are food costs a barrier? Public Health Nutr. (2011) 15(2):370–6. 10.1017/S1368980011002382 22014448 PMC4447204

[61] Monsivais P Kirkpatrick S Johnson DB. More nutritious food is served in child-care homes receiving higher federal food subsidies. J Am Diet Assoc. (2011) 111(5):721–6. 10.1016/j.jada.2011.02.007 21515119

[62] Kenney EL Tucker K Plummer RS Mita C Andreyeva T. The child and adult care food program and young children’s health: a systematic review. Nutr Rev. (2023) 81(11):1402–13. 10.1093/nutrit/nuad016. 36882043 PMC10563858

[63] Dutko P Ver Ploeg M Farrigan T. Characteristics and influential factors of food deserts (2012). [Report] Economic research report number 140. p. 36. 10.22004/ag.econ.262229

[64] Hardin-Fanning F Rayens MK. Food cost disparities in rural communities. Health Promot Pract. (2014) 16(3):383–91. 10.1177/1524839914554454 25305093 PMC4393346

